# Exploring the utility of robots in exposure studies

**DOI:** 10.1038/s41370-019-0190-x

**Published:** 2019-11-19

**Authors:** Elisabeth Feld-Cook, Rahul Shome, Rosemary T. Zaleski, Krishnan Mohan, Hristiyan Kourtev, Kostas E. Bekris, Clifford P. Weisel, Jennifer (Mi K.) Shin

**Affiliations:** 1 Environmental and Occupational Health Sciences Institute, Rutgers, The State University of New Jersey, Piscataway, NJ 08854 USA; 2 PRACSYS Lab, Department of Computer Science, School of Arts and Sciences at Rutgers, The State University of New Jersey, Piscataway, NJ 08854 USA; 3 ExxonMobil Biomedical Sciences Inc., Annandale, NJ 08801 USA; 4 ExxonMobil Biomedical Sciences Inc., Spring, TX 77389 USA

**Keywords:** Personal exposure, Exposure modeling, Volatile organic compounds, Robots

## Abstract

Obtaining valid, reliable quantitative exposure data can be a significant challenge for industrial hygienists, exposure scientists, and other health science professionals. In this proof-of-concept study, a robotic platform was programmed to perform a simple task as a plausible alternative to human subjects in exposure studies for generating exposure data. The use of robots offers several advantages over the use of humans. Research can be completed more efficiently and there is no need to recruit, screen, or train volunteers. In addition, robots can perform tasks repeatedly without getting tired allowing for collection of an unlimited number of measurements using different chemicals to assess exposure impacts from formulation changes and new product development. The use of robots also eliminates concerns with intentional human exposures while removing health research ethics review requirements which are time consuming. In this study, a humanoid robot was programmed to paint drywall, while volatile organic compounds were measured in air for comparison to model estimates. The measured air concentrations generally agreed with more advanced exposure model estimates. These findings suggest that robots have potential as a methodology for generating exposure measurements relevant to human activities, but without using human subjects.

## Introduction

Robot use is increasing in the workplace and throughout society in general [1, 2]. The complexity of tasks (any scenario the robot executes) performed by robots continues to grow, and as more affordable, functional robots become available, there are increasingly more routine applications that robots can perform at home, in the workplace, for emergency response activities, research, therapy, and mundane day to day tasks [2–4]. Within occupational settings, like the food industry, robots have been designed and programmed to perform specific tasks to promote the health and safety of workers [1, 5]. Nevertheless, robots have had limited use in exposure science research, such as estimating airbourne particle exposures [6, 7] and simulating infant behavior to obtain a more accurate indoor infant exposure profile due to the challenges in sample collection from this age group [8–13]. With the rapid and accelerating advancement in robotic technology, a proof of concept study was designed to determine the utility of robots to perform activities that lead to exposure and to facilitate the collection of air samples to estimate inhalation exposure.

The primary focus of the study was to determine if robots can be used as a new methodology for generating exposure data as a plausible alternative to employing human subjects in exposure studies. If robots are effective in this domain, as indicated by this study, they can help close exposure knowledge gaps where data are:

- Currently lacking (e.g., new products or formulations, infrequent tasks)
- Needed as specific inputs for exposure models
- Or difficult to acquire (e.g., consumer product safety)

This robotic-based e-methodology would be very useful where staged simulations may have ethical considerations related to intentional exposure or when repetitive actions are desired to evaluate the distribution of exposures. Human-based studies are subject to institutional review board (IRB) protocols and approval requirements, as well as considerations to data privacy laws related to the confidentiality of human subjects. Developing a way to address these issues can save time, facilitate changes in protocols while the studies are on-going, and reduce administrative overhead needed for tracking subjects and outcomes, as well as justifying risk-benefit ratios for intentional exposures. Robots do not have to adhere to the aforementioned considerations and requirements. They have the ability to complement human subject exposure studies by providing exposure ranges and uncertainty estimates for specified activities thereby advancing the field of exposure science.

This study explored the feasibility of using a robot in place of humans to generate exposure data for exposure estimation. In addition, the measured exposure profile, specifically of air concentrations during robotic activity, was evaluated by comparison to model estimates.

## Materials and methods

### Exposure chamber and monitor setup

A dual-arm humanoid robotic platform was programmed to perform a simple task of painting drywall. Painting was chosen because of the readily available consumer products (i.e., paint) and manageable necessary programming to generate painting motions using a roller. One type of low-VOC water-based paint (WBP) was used as received throughout the study. All painting trials (daily robot activity including setup and postpainting sampling) performed in this study were executed inside Rutgers University’s Controlled Environmental Facility (CEF) to ensure uniform conditions (i.e., temperature and humidity) throughout the study. During each of the six painting trials (A–F), short term (trial duration) and long term (8 h from the start of painting) air samples were collected using a combination of personal volatile organic compound (VOC) real-time monitors, thermal desorption (TD) tubes, and consumer indoor air quality (IAQ) monitors^1^. Other direct reading instruments with data logging, for measuring total hydrocarbons, relative humidity, and temperature were collocated for 24 h of continuous measurement during and after the trial completion.

**Fig. 1:**
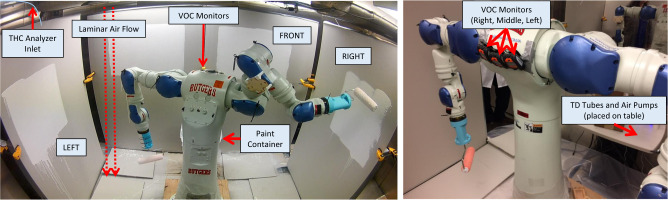
A real-time photo of the inside of the CEF during the robotic painting from the back (left) shows the placement of the drywall, robot, paint, and the THC analyzer sampling inlet. A photo from the front of the CEF (right) shows the placement of the VOC monitors and TD tubes. The distance between the robot and each drywall panel is 1.17 m in front and 1.2 m on each side

Prior to the start of the first painting trial, continuous monitoring was conducted using the total hydrocarbon (THC) analyzer for 24 h to evaluate the release of VOCs from the drywall. Prior to and upon completion of each painting trial, the pre/post mass of paint and the length and width of the painted drywall area was measured and recorded. The temperature and relative humidity were kept at 25 ± 0.46 °C and 40 ± 6%, respectively. Four new ultralight gypsum drywall panels were placed inside the chamber before each painting trial as described in Fig. 1. Each drywall panel was trimmed to measure 4′ × 6′ × ½″.

### Robotic platform

**Fig. 2:**
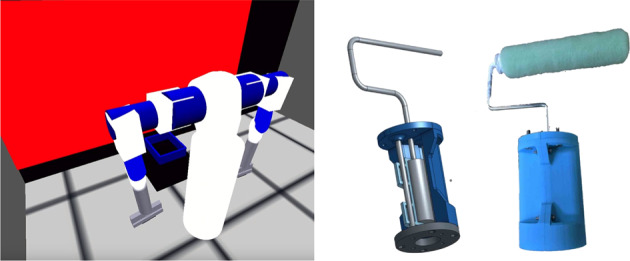
*Left*: a simulation environment was developed by the research team to replicate the real-world experimental setup in the measurement chamber. The figure shows the simulator’s visualization, where the robot manipulator is in its initial configuration with the compliant paint-roller attached. The simulation also includes the positions of the paint container and dry wall boards (shown in red). The robot’s motion is first programmed and tested in simulation for safety and effectiveness before deployed in the real setup. Accurate-enough reproductions of the geometries of the robot, the paint roller, the paint bucket and the walls, as well as corresponding software, are needed to produce motions that **a** avoid undesirable collisions, and **b** result in contact between the roller geometry and the target wall. *Right*: the figure shows the 3D digital model of the specially designed compliant paint-roller, which was attached to the robotic arm, right next to the real one. The real system was constructed from 3D printed components based on the digital model. The key feature of the paint-roller is that it has an internal spring-loaded mechanism—highlighted in the digital model—which provides compliance and robustness to positioning errors. This makes it possible to use the robot for the intended purpose without the need for expensive sensors and a complex sensor-monitoring process

The experiments were performed using a Yaskawa (Kitakyushu, Fukuoka Prefecture, Japan) Motoman SDA10F bimanual robotic manipulator with seven joints in each arm, and a single revolute joint at the robot’s torso. The torsional degree of freedom allows the robot to rotate its arms and provides increased reachability. In order to apply paint on the wall at a realistic rate with minimal requirements in terms of computational reasoning and sensory information, a novel robotic end-effector was designed with a spring-loaded mechanism connected to a standard paint-roller (Fig. 2, right). The physically adaptive nature of the spring-loaded mechanism allows the necessary pressure to be applied on the drywall in a sensor-less manner without damaging the robot or the drywall. The entire experimental setup was first generated in simulation (Fig. 2, left). The robot and drywall were modeled in the simulation environment and motions were computed using a motion planning software framework [4]. The objective of this process was to generate motions that maximized the area of drywall covered by paint, using human-like sweeping motions over contiguous vertical planes in front, and to the side of each arm. Motions were also generated to dip the paint-rollers into the paint container. All generated motions are disallowed from colliding with any object in the simulated scene to ensure that the robot does not risk damaging the setup. Once the motions were generated in simulation, they were transferred and subsequently executed by the real-robot in the CEF. Repeated identical painting motions were performed for the duration of the trials, involving both arms, one at a time, painting the drywall panels in front of it and to its side.

### Painting trial parameters

During each painting trial, the robot performed seven painting cycles (set of generated painting motions) over the same drywall area for all trials except the first, where it performed ten cycles, for an average of 53 ± 6.2 min at a high air exchange rate (AER) of 11–12 h^−1^ or at a low AER of 6–8.5 h^−1^. AER was determined by measurements of the decrease of CO_2_ added to the CEF at an initial concentration of 1900 ppm (Table 1). The AER are consistent with the AER found in buildings when the windows are open to provide appropriate ventilation during painting [14].

**Table 1:** Painting task parameters throughout the study

	Trial A	Trial B	Trial C	Trial D	Trial E	Trial F	Average
Painting duration	66 min	50 min	52 min	51 min	49 min	47 min	53 ± 6.2 min
Amount of paint used	2.4 kg	1.3 kg	1.5 kg	1.8 kg	1.6 kg	1.8 kg	1.53 ± 0.64 kg
Measured air exchange rate^a^	11–12	11–12	11–12	6	8.5	8	

^a^Measured using CO_2_ gas flow

At the beginning of each painting cycle, the roller was dipped into the paint for 5 s then held for 1 min at a 45° angle over the paint container to let the extra paint drip off before it touched the drywall. Next, a sequence of four generated motions: first, the left arm painting in front, next, the left arm painting to the left, then, the right arm painting in front, and lastly, the right arm painting to the right (Fig. 1). A dry run-through, consisting of the robot performing one cycle of the generated motions, was conducted before each trial to adjust the placement of boards to assure the robot touched the surface of the drywall with the roller.

### Exposure models

In order to evaluate the robotic methodology, paint exposures were estimated using a range of consumer and worker modeling tools including models covering low tier (most conservative) to high tier (most realistic) exposure predictions. Lower tier models are easy to use and are based on readily available data; however they are usually built on conservative assumptions and typically overestimate exposures. Higher tier models can be more difficult to use, but they can reduce or quantify uncertainty.

Models used for this study include: European Solvents Industry Group (ESIG) Generic Exposure Scenario (GES) Risk and Exposure Tool (EGRET) [15], Targeted Risk Assessment Tool (TRA) [16], Consumer Exposure Models (ConsExpo) [17], Exposure & Fate Assessment Screening Tool (EFAST) [18], Well Mixed Box Model Constant Generation Rate (WMB) [19], and Advanced Reach Tool (ART) [20]. These models were used to estimate average VOC air concentrations for the duration of the trial for three separate trials.

Models were run to approximate conditions for three specific trials, selected to span the range of measured results over the trials. The trials selected included the days with maximum and minimum amount of paint used under the higher ventilation conditions (Trials A and B), and the maximum amount of paint used under the lower ventilation conditions (Trial D). Not all parameters in the low tier models were adjustable. Higher tier models were set to match the experimental conditions as closely as possible. Detailed information on the tools, the assumptions used, model inputs, and model outputs are included in the supplemental material (Supplemental Material 1).

### Total hydrocarbon analyzer

A Thermo Scientific™ (Waltham, MA, USA) 51i THC analyzer was used to measure the concentration of total nonmethane hydrocarbons present throughout the duration of the study. The THC analyzer records data points averaged over 60 s intervals and responds to a wide range of volatile compounds, including VOCs off-gassing from paint. The THC analyzer uses a flame ionization detector to detect organic compounds via combustion with a hydrogen flame [21] and was calibrated with propane. The THC analyzer inlet was located in the back of the chamber at the top of the ceiling, behind the robot (Fig. 1). The observed background readings for the THC analyzer were generally <0.1 ppm and did not exceed 0.2 ppm.

### VOC monitors

Ion Science (Fowlmere, Royston, UK) CUB monitors [22] were used as personal VOC monitors to detect the concentration of VOCs in the general “breathing zone” area of the robot during short term (trial duration) and long term (8 h) durations. A measurement reading was taken approximately every 20 s. These VOC monitors use a photoionization detector (PID) to detect compounds with a part-per-billion (ppb) limit of detection. The PID response varies with compound molecular structure and functional group and is most sensitive to aromatics and olefins [22]. It is important to note this limitation since the VOCs of interest have a range of structures which can account for some of the differences in the air concentrations measured by multiple instruments shown in the results section [22]. Three VOC monitors were placed on the front of the robot in-between the arms on the center of the robot’s body that rotated with movement (Fig. 1). The personal VOC monitors were calibrated before and after each trial with isobutylene.

### Thermal desorption tubes

The thermal desorption tubes (TD tubes) are active air samplers used to collect VOCs during the painting trials for subsequent Gas Chromatography/Mass Spectrometry (GC/MS) analysis. Two multibed (graphitized carbon black and carbon molecular sieve adsorbents), carbotrap 300 equivalent TD (Supelco, Bellefonte, PA, USA) were attached to an air pump and set on a ~3 foot high table in the back of the CEF behind the robot (Fig. 1). The air pumps were set to an average flow rate of 60.5 cc/min for all trials except the first, which had a flow rate of 30 cc/min. The total collection time was equivalent to the duration of the painting trial (short term), in order to collect an average of 3.42 ± 0.73 L of air, sufficient to detect organic compounds via GC/MS at expected concentrations of low µg/m^3^. Quantitative results were not used for the statistical analysis because the calibration mix of the EPA TO-17 method did not have the compounds coming from the paint. The qualitative results were used for comparison with a GC/MS headspace analysis (Supplemental Material 2) of the paint to confirm what compounds were attributed to the paint. These compounds were then used in the model estimates.

### Data analysis

Temperature and relative humidity data were continually collected. The TD tubes were analyzed using EPA method TO-17 for determining toxic organic compounds, on a GC/MS to verify the identity of compounds emitted from low-VOC WBP. The chromatogram peaks that increased during painting were identified through a match of the mass spectrum to a NIST library (Wiley 1.01, 9th edition). Background measurements were collected pre- and postpainting for each trial. Background subtraction was performed for both the THC analyzer and VOC monitors. To accurately determine the background for the THC analyzer and VOC monitor data, an average of each dataset collected 10 min prior to the start time of each individual painting trial was used as the background for that trial. This calculation accounted for factors that could potentially change the background concentration throughout the study. The VOC monitor and THC analyzer data were normalized to the amount of paint used in each trial.

As stated previously, the direct-read, real-time monitors used in this study do not have equivalent responses to all categories of VOCs and were calibrated with different gases. In order to compare the datasets, all the VOC monitor and THC analyzer data were converted to ppm per carbon (ppm-C) as methane equivalents. For the VOC monitors, isobutylene (4-carbon) was used for calibration so the data were multiplied by four to get the ppm-C as methane equivalent values. For the THC analyzer, the data was multiplied by three to convert to ppm-C as methane equivalents because propane (3-carbon) was used for calibration. While this conversion allows for a better comparison of the data between monitors, it does not account for the differential monitor responses to diverse classes of compounds. This limitation is explored further in the discussion section.

Since few personal exposure measurements during painting with low-VOC WBP were identified in the literature, our experimental results were compared with model estimates to help determine if exposure data collected during the robotic painting are representative of the exposure during human painting. To compare measured data to model estimates, VOC air concentrations were converted from ppm-C to mg/m^3^ using the average molecular weight (MW) of compounds detected in the headspace analysis of the WBP and the ratio of the total atomic weight of carbons in the compound, W_c_, to MW. For example, the molecular weight (MW_total_) of butyl propionate is 130 g/mol; there are seven carbons, so the total weight of carbon in the compound (W_c_) is 84 g/mol. Calculating the ratio of total MW_total_ to W_c_ (130/84 g/mol) results in 1.55. The average ratio of the four compounds identified as coming from the paint detected in the headspace analysis (Table 2) is ~1.5, resulting in a final conversion formula shown below:

**Table 2:** Compounds detected in headspace analysis of WBP

	MW (g/mol)	Formula	Ratio of total MW to C MW
Methyl methacrylate	100	C_5_H_8_O_2_	1.7
n-butyl ether	130	C_8_H_18_0	1.4
Butyl acetate	116	C_6_H_12_0_2_	1.6
Butyl propionate	130	C_7_H_14_O_2_	1.5
**Average**	**119**	–	**1.5**

Standard conversion formula for ppm to mg/m^3^ based on 25 °C and 1 atm.^2^:

1 $$Y \,=\, \frac{{X\left( {\rm{Total}}\,\,{\rm{Molecular}}\,\,{\rm{Weight}} \right)}}{{24.45}}$$

where the units of *Y* and *X* are the concentration in mg/m^3^ and ppm, respectively.

Conversion formula for average VOC air concentration using headspace analysis result:

2 $$Y =	 \, \frac{{X\left( {\rm{AW}}_{\rm{c}}\, {\rm{\times}}\, \left( {\rm{ratio}}\,{\rm{of}}\,{\rm{MW}}_{\rm{total}}\,{\rm{to}}\,{W}_{c} \right) \right)}}{24.45}\\ =	 \, \frac{X(12\,{\rm{\times}}\,1.5)}{24.45}\\ =	 \, X\,{\rm{\times}}\,0.74$$

where AW_c_ is the atomic mass of carbon, 12 g/mol, MW_total_ is the compound molecular weight, *W*_c_ is the total weight of carbon in the compound, *X* is the VOC air concentration in ppm-C and *Y* is the same as Eq. (1).

Table 2 outlines the parameters used in the calculation (described in Eqs. (1)–(2)) to determine the vapor pressure (VP) of each compound found in WBP. The VP and MW of the compounds were used in the models.

## Results and discussion

### Exposure evaluation

VOCs encompass a large number of carbon-containing compounds spanning multiple chemical classes, have been linked to acute and/or chronic health effects, and can be released as vapors from a variety of products and materials including paints [23, 24]. Some VOCs have been linked to acute and/or chronic health effects, such as headaches, respiratory tract and eye irritation, liver and kidney damage, and some are known carcinogens [23, 24]. Low-VOC paint can contain up to 50 g/L of VOCs [25]; the VOCs commonly found in WBP are ethylene glycol, texanol, and propylene glycols [26–29].

**Fig. 3:**
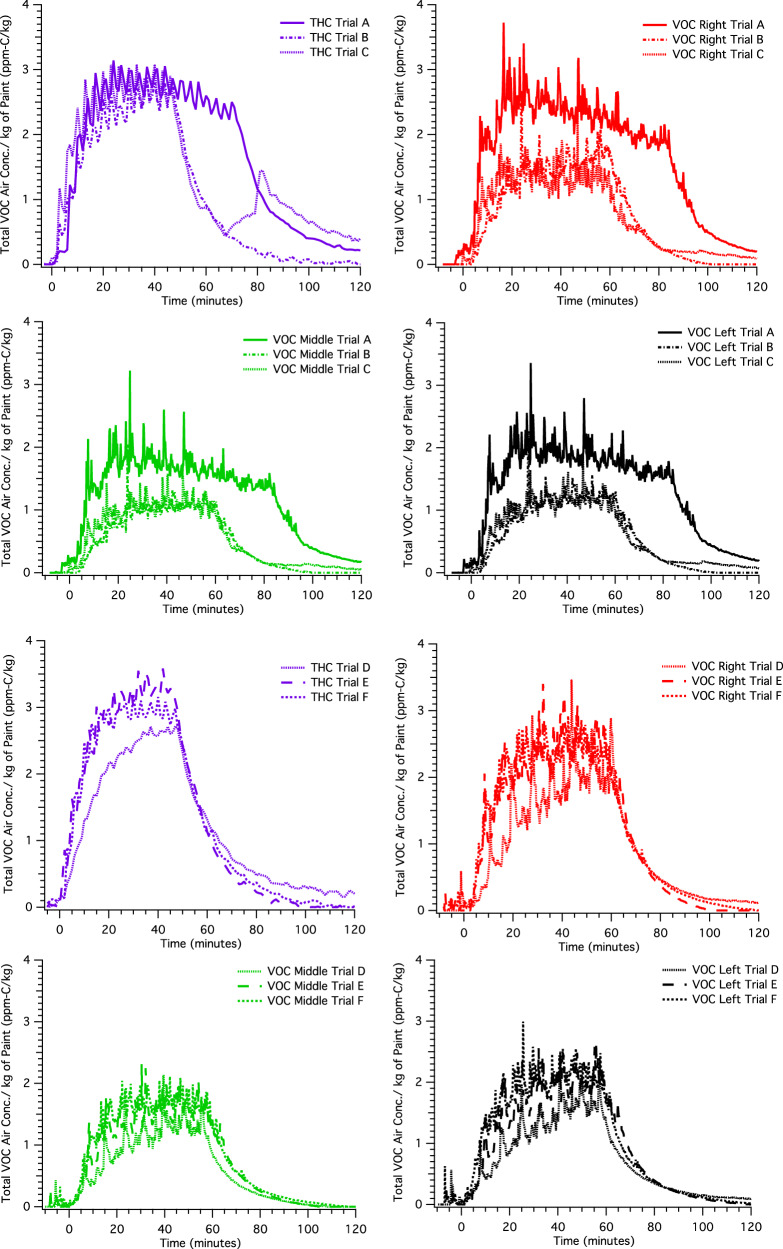
Real-time THC and VOC monitor data normalized to the amount of paint used in each trial are shown for the painting trial (short term) for trials **a**–**c** with the high ACH (top four graphs) and trials **d**–**f** with the low ACH (bottom four graphs)

The results for VOC air concentrations during the painting trial for the THC analyzer and the personal VOC monitors are summarized in Table 3. The background measurements for the THC and VOC monitors, temperature, and relative humidity indicate that the controlled environment had a background VOC air concentration of <0.2 ppm-C [30–32] (Supplemental Material 2: Fig. S1). Temperature and humidity were kept constant (Supplementary Material 2: Figs. S1, S4–6, S9–11). The THC analyzer measured the highest air concentrations, even though it was farther away from the paint and drywall compared with the VOC monitors, most likely due to its higher response to saturated and oxygenated hydrocarbons than the PID deployed in the VOC monitors (Fig. 3, Table 3). The THC analyzer and VOC monitors measured the same trends in VOC air concentrations during (Fig. 3) and after (Supplemental Material 2: Figs. S2, 3, S7, 8) the painting trial. The VOC air concentration rose rapidly at the beginning of the trial, leveling off within 15–20 min after the painting started and remaining nearly constant for the duration of the painting. Once the painting was completed and the unused paint was removed, the air concentration declined in an exponential fashion returning to background levels within 2 or 3 h. The periodicity in the air concentration during the painting may be due to the robot obtaining more paint to conduct a new paint stroke. The same trend was observed for all seven painting trials (Fig. 3, Supplemental Material 2: S2, 3, S7, 8).

**Table 3:** The average VOC air concentrations measured during the painting task (short term)

	THC analyzer^a^	VOC Monitor *Right*^a^	VOC Monitor *Middle*^a^	VOC Monitor *Left*^a^
Trial A	5.95 ± 1.56	5.22 ± 1.43	3.77 ± 1.02	4.15 ± 1.14
Trial B	2.52 ± 1.00	1.56 ± 0.68	1.05 ± 0.46	1.18 ± 0.52
Trial C	3.46 ± 1.14	1.88 ± 0.54	1.45 ± 0.44	1.54 ± 0.47
Trial D	2.97 ± 1.23	2.70 ± 1.31	1.68 ± 0.85	1.98 ± 1.00
Trial E	4.27 ± 1.42	3.23 ± 1.27	2.11 ± 0.82	2.59 ± 1.03
Trial F	4.47 ± 1.37	3.57 ± 1.27	2.52 ± 0.88	3.10 ± 1.13

^a^Concentrations are background corrected and reported as ppm-C

**Fig. 4:**
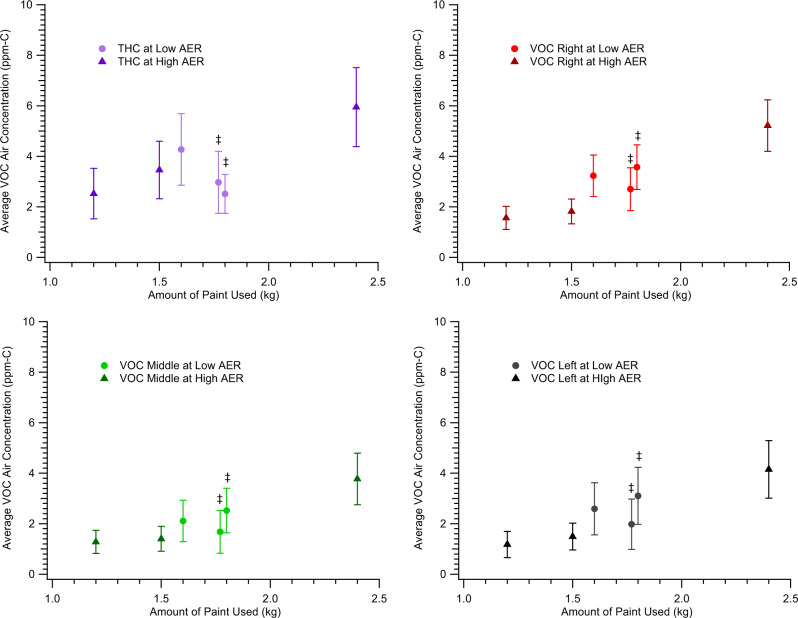
Average VOC air concentrations across the painting time for the THC (top left) and all three VOC monitors (top right, bottom left and right). Trials performed at higher AER are triangles and lower AER are circles. The errors bars represent the standard deviation. ^‡^Two trials used exactly the same amount of paint (1.8 kg) and are separated for clarity

A strong correlation, with *R*^2^ values of 0.89, 0.86, 0.85 for the right, middle, and left VOC monitors, respectively, was observed, showing an increasing trend for the average VOC air concentration per amount of paint used (Fig. 4). The *R*^2^ value for the THC analyzer was 0.56, indicating less of a correlation between the average VOC air concentrations and amount of paint (Fig. 4) compared with the VOC monitors. This is most likely due to a combination of the laminar flow of air within the CEF, its differential response to compound classes, and proximity to the painted drywall. However, at high AER, the VOC air concentrations for the THC analyzer show an increasing trend with increasing amount of paint. The same increase is observed for the VOC monitors at high AER. The average VOC air concentrations at low AER for both the THC analyzer and VOC monitors are clustered together and do not visually show a distinct pattern. This phenomenon could be due to more mixing at the higher AER.

Five major volatile components were identified based on comparison of a library mass spectral match in the head space above the paint: acetone, methyl methacrylate, butyl acetate, n-butyl ether, and butyl propionate. While all five were present in the TD tubes, acetone was also present in background samples in high concentrations. The four other compounds were found in the TD tube samples collected during the painting trials but were absent in the background and blank TD tube samples. Thus, the average VP and MW of these four compounds were used in the models to estimate VOC air concentrations for three separate trials as described in the next section.

### Model estimates

Since limited exposure data using low-VOC WBP have been reported in the literature and those reported are dependent on the painting conditions, a series of mathematical models were run, using a tiered approach, for comparison to experimental measurements. Lower tier models were run first, as this was a proof of concept study looking at the use of robots for exposure data generation. Higher tier models were then run using more of the experimental conditions to provide more refined estimates.

**Fig. 5:**
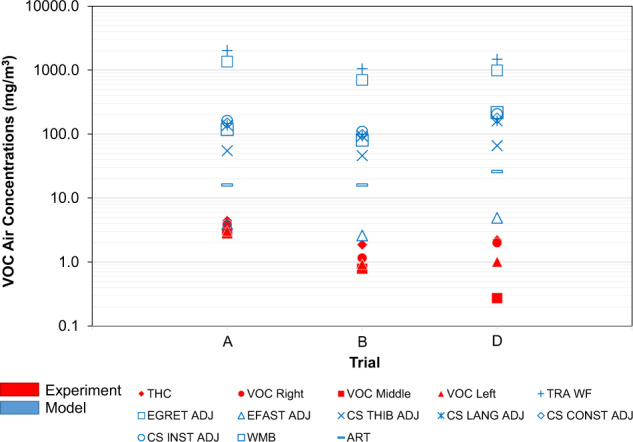
Comparison of model estimates to measured data

The model estimates and the measured results from the THC analyzer and VOC monitors are summarized in Table 4 and Fig. 5. In general, the models used in this study provided expected results based on their respective tiers. Lower tier models (TRA, EGRET, CS instantaneous) overestimated VOC concentrations, mid-tier tools had less overestimation (CS constant rate, CS evaporation Langmuir isotherm, WMB), and higher tier model estimates were within the range of measured concentrations by ~1 order of magnitude (CS evaporation Thibodeaux E-FAST wall paint specific algorithm, ART).

**Table 4:** Comparison of measured results and model estimates for Trials A, B, and D

		Trial A (mg/m^3^)	Trial B (mg/m^3^)	Trial D (mg/m^3^)
Measured air concentrations	THC analyzer	4.4	1.9	2.2
	VOC monitor right	3.9	1.2	2.0
	VOC monitor middle	2.8	0.8	0.3
	VOC monitor left	3.1	0.9	1.0
Model estimates	TRA	2032	1049	1483
	EGRET	1354	699	988
	WMB	118	80.6	217
	CS INST (instantaneous rate)	161	109	205
	CS CONST (constant rate)	148	98	177
	CS LANG (evaporation, Langmuir isotherm)	137	93	160
	CS THIB (evaporation, Thibodeaux isotherm)	55	46	66
	EFAST	3.9	2.6	4.9
	ART (median)	16	16	26

*TRA* targeted risk assessment tool, *EGRET* European Solvents Industry Group (ESIG) Generic Exposure Scenario (GES) Risk and Exposure Tool, *WMB* well mixed box model constant generation rate, *CS* consumer exposure models (also known as ConsExpo), *EFAST* exposure and fate assessment screening tool, *ART* advanced reach tool

### Evaluating the utility of robotics technology for exposure studies

The average measurements of the resulting painted areas for all trials are shown in Table 5. The complete set of measurements for each trial are shown in Supplementary Material 2: Table S1. As expected, the area painted by the robot was highly repetitive with standard deviations within 1–2 cm for each drywall panel. The amount of paint used for trials B–F was 1.3–1.8 kg but trial A was performed for a longer period of time so it used more paint (2.4 kg), which resulted in excess dripping. This issue was corrected in later trials by reducing the duration of painting and therefore the amount of paint applied. The variation observed for trials B–F is likely due to small differences in pressure applied to the roller while painting, slight changes in how much paint dripped off the roller prior to touching the drywall during the trial, and the two different air exchange rates used. The differences in the width on different drywall panels can be attributed to the robot’s reachability region, given the placement of the drywall panels in front of it and to its side. The software aimed to maximize the region on each drywall panel that was painted by the robot, given the CEF dimensions and the robot’s reachability. The setup allowed for more space (width) in the front than on the sides of the robot. The robot was able to rotate to paint the side panel but not able to bend down at the waist. The robot painted an average width within 9 cm and the height within 1 cm for 28 drywall panels. Even though the robot did not have pressure sensors on the arms, approximately the same amount of pressure was applied by the arm during painting. However, the pressure of the roller could have changed due to minor differences in the distance between the robot and drywall. This is not unlike human painters who likely apply different pressure to the roller to achieve complete coverage of paint. More sophisticated robotic setups could utilize sensors to minimize pressure changes. This would reduce the variability in the amount of paint applied and allow for simulation of a wider range of human behaviors while performing specific tasks to more accurately evaluate how they might alter exposure.

**Table 5:** Average painted area measurements for all drywall used for all trials

Painting area	Average height (cm)	Average width (cm)	Average area (cm^2^)	Coefficient of variation (CV)
Front left	58 ± 1	78 ± 1	4531 ± 61	1.3
Left	60 ± 2	63 ± 2	3765 ± 63	1.7
Front right	60 ± 2	77 ± 1	4654 ± 173	3.7
Right	60 ± 1	58 ± 2	3441 ± 111	3.2
Total	59 ± 1	69 ± 9	4093 ± 534	13

In the current setup, care was taken to place the drywall panels on stable frames at the target distance from the robot. The motions, originally generated in simulation, were replayed in the real-world experimental setup. No sensing was used to adapt the motions to any minor variations of the drywall placement between experiments. The physically adaptive nature of the paint roller accounted for the lack of sensing and ensured application of paint on all the surfaces across all the experimental runs.

During the study, some advantages and disadvantages of using robots for exposure estimation were identified. The chief advantages are (i) safety, (ii) repeatability of the activities and resulting exposures, and (iii) a robot’s ability to be programmed to test a large set of predetermined variations in behavior, in the context of repetitive tasks. The use of robots instead of human subjects eliminates intentional exposures to emissions during the activities of staged exposure studies, thus eliminating the need for full IRB involvement and privacy laws related to human subject considerations. Modern robot manipulators, like the one used in this study, provide accurate repeatable motions within a few millimeters. In particular, a robot allows the experiment to be replicated with a high degree of fidelity, allowing for a precise evaluation of how various conditions can alter exposures. In addition, this study aims to encourage the development and deployment of specially designed robotic platforms for exposure studies. This will allow the precise control of the robotic movement and sensing of its environment so that researchers can easily define different types of behaviors and measure any variability that might be of interest in a controlled manner (e.g., variations between different human subjects such as pressure and amount of paint). Further, a robotic platform has no limit on the number of samples and repetitions performed in a uniform fashion, a limitation of using human studies brought on by general fatigue of the subjects.

The type of actions that can be included and measured in the experiment is limited by the capabilities of the robot and the setup. For instance, a mobile robot could paint large stretches of walls compared to a stationary model that can reach only its immediate surroundings. In this study, the portion of painted drywall was less than originally anticipated due to the lack of bending capabilities in the robot’s waist. The kind of motions that a robot can perform are unique to the specific hardware platform. Careful choice of the robot’s design and setup must be made to ensure a comparable and faithful emulation of a human doing the same task.

Even though a robot provides significant advantages, there is an overhead cost associated with setting up the experiment. At this point in time, specialized robotics operators and programmers are required to design the motions. When deploying robots to truly unstructured scenes for more complex studies, sophisticated sensing and planning abilities are required because the task can become arbitrarily complex for the robot. Robust sensing and vision systems might be required to make the robots adapt to changes in the environment. Moreover, robotic platforms and logistical support might not be readily available to all researchers currently. The above issues are quickly changing through the introduction of increasingly affordable and capable robots to be made available for research and industrial use. Furthermore, robot control interfaces are being increasingly simplified to allow for the use of robots by nonexperts.

## Conclusion

There is a convergence of developments in robotics, which allows the adaptation of this study’s methodology to a wide variety of tasks. The types of exposure scenarios that can be evaluated using this approach will increase as the field of robotics advances. For example, exposure data generated by robots can be used to assess tasks in which the outcome depends on how the worker or consumer performs the task (e.g., welding, painting, cleaning, and spraying). This approach can also be used to evaluate the impacts to exposures from the use of different substances in the same setup (e.g., new formulations or products), or impacts from other exposure determinants (e.g., controls, secondary sources). Examples of key technologies may include: activity recognition of human actions from visual data and mapping of the corresponding operations to human motion; learning from demonstration, where robots are trained by humans in order to replicate specific tasks; and tactile sensors placed on human hands, which can provide high-fidelity data for delicate operations performed by people that can assist the mapping of the motion to robots.

The current study provides a working motivation for using robotic platforms in exposure studies, especially to fill exposure data gaps mentioned above. The reproducibility and applicability of the robot were demonstrated through a simple task: painting drywall. The potential exposure estimates generated from the robotic platform are consistent with higher-tiered modeled estimates for painting. With advances in the capabilities of robotic platforms, and their ubiquitous and affordable availability, it is expected that robots will provide a safe and reliable platform for exposure estimation in the future.

## Supplementary information

Supplementary Material 1

Supplementary Material 2

## Footnotes

1 The results of the consumer monitors will be discussed in a future publication.

2 https://www.cdc.gov/niosh/docs/2004-101/calc.html
