# Supplementary material for: Exploring the utility of robots in exposure studies
Source: J Expo Sci Environ Epidemiol. 2019 Nov 19;31(4):784–94. doi: 10.1038/s41370-019-0190-x (PMC7234925; doi:10.1038/s41370-019-0190-x)
Supplement: Supplementary file 1 — Supplementary Material 1 [file 41370_2019_190_MOESM1_ESM.docx]

**Supplemental Material 1**

Several commonly used consumer and worker exposure modeling tools were used to help evaluate how well the exposures measured during the robot painting event would match those predicted for human exposure during painting.

The tools were selected to span modeling tiers, with lower tier models intentionally designed to be conservative (i.e., over-predict rather than under-predict exposure) and higher tier models that enable additional refinement of the predicted exposure to provide more realistic estimates.

The tools selected included:

Consumer Exposure Tools

- ECETOC TRA: European Center for Ecotoxicology and Toxicology of Chemicals Targeted Risk Assessment Tool, a Tier 1 (lowest tier) screening level model.
- EGRET: European Solvent Industry Group’s Generic Exposure Scenario Risk and Exposure Tool, a Tier 1.5 screening model, based upon the TRA but with additional refinements.
- ConsExpo: Consumer Exposure model of RIVM, the Netherlands Institute for Public Health and the Environment, can be used as a higher tier to lower tier model depending upon the algorithms chosen. The lowest tier is the instantaneous release algorithm which corresponds to the algorithm in the TRA and EGRET models. Increasingly higher tiers are the constant release algorithm with evaporation being the highest.
- EFAST: Exposure and Fate Assessment Tool, USEPA exposure model, painting module is based upon the Wall Paint Exposure Model (WPEM), a model specifically designed for wall painting and already evaluated with measured data. EPA lists this as a screening level model but it is more advanced than the other lower tier tools used here, such as multiple zones within a house. In addition, the wall paint algorithm, as mentioned is based upon and evaluated against actual measurements of wall painting exposure.

Worker Exposure Tools

- WMB: Well-Mixed Box Model with a Constant Emission Rate (using IH MOD 2.0), a simple, lower tier model and assumes that the contaminant concentration in a room is well mixed due to either natural or induced air currents. This model provides a quick first level assessment.
- ART: Advanced Reach Tool 1.5, is a tier 2 exposure assessment tool that combines a mechanistic model with functionality to update model estimates with measurements (selected from a built-in exposure database or user’s measurements) using a Bayesian approach.

To develop estimates of air concentrations, all of the consumer models require vapor pressure (screening models only require knowing a vapor pressure (VP) band, in this case is it greater than 10 Pa, higher tier models require a specific number), some require molecular weight (MW) (ConsExpo and E-FAST; TRA and EGRET if saturated vapor concentration will be used as an upper bound air concentration), and running ConsExpo in the most refined mode requires Log Kow (octanol-water partition coefficient). For the worker exposure tools, the well-mixed room model did not require the vapor pressure while ART 1.5 required a specific vapor pressure.

In addition, exposure models require a weight fraction of the constituent being modeled. In some cases default values are included. To adjust modeling conditions as closely as possible to the paint trials a weight fraction of 0.02, equal to the maximum allowable VOC content in a low VOC paint, was used. The actual VOC content could have been lower than this value. “Zero-VOC” paints have a cutoff of 0.005 g, and so the actual VOC content of the low VOC paint likely fell within the 0.005 – 0.02 range.

In order to approximate these values, a headspace analysis from another container of the same brand, color and type of low VOC paint was run. Four constituents were identified. The average MW, VP and log Kow for these 4 constituents was used as inputs for the models when these parameters were needed (Table 1). Note, one additional constituent was detected in the headspace but it was also detected in the blank sample, therefore it was excluded from this analysis. If it had been included in the average, it would have had little impact on the MW or ratio of total MW to weight of Carbon per mole, but would have increased the total VP. This would not have impacted the TRA or EGRET estimates which are already fall in the highest VP band for these models, but it would have resulted in higher predicted air concentrations for the higher tier modeling. We recognize that the approach of averaging values across headspace constituents consistent with paint components provides some uncertainty into the model analysis, but feel the use of the average values was a sufficient level of resolution for the purposes of this proof of concept project, i.e., understanding if measured values were consistent with model predictions for human exposure.

| Table S1. Properties of Paint Head Space Constituents | | | | |
| --- | --- | --- | --- | --- |
| **Constituent** | **Vapor Pressure (in Pascals)** | **Molecular Weight** | **Number of Carbons** | **Octanol Water Partition Coefficient (Log Kow)** |
| methyl methacrylate | 5133 | 100 | 5 | 1.38 |
| n butyl ether | 800 | 130 | 8 | 3.21 |
| butyl acetate | 1533 | 116 | 6 | 1.78 |
| butyl propionate | 589 | 130 | 7 | 2.10 |
| **Average** | **2014** | **119** | **6.5** | **2.12** |

Models were run to approximate conditions for three specific trials, selected to span the range of exposures over the low VOC paint trials. The days selected included the days with maximum and minimum amount of paint used under the higher ventilation conditions (Trials A and B), and the maximum amount of paint used under the lower ventilation conditions (Trial D). Results are provided for the screening level models (TRA and EGRET) using all defaults and also with adjustable parameters set to match the experimental conditions. Not all parameters in the screening level models were adjustable. Higher tier models were set to match the experimental conditions as closely as possible. ConsExpo can produce both lower tier estimates (instantaneous mode) and higher tier (constant rate or 2 options for evaporation mode), all of these options were included. Results for the mean air concentration during the period of painting (mean event concentration) and information on the approach taken for each model is detailed below. Modifications to model defaults values are specified below and summarized in Table S2.

| Table S2. Model Predictions – mean event concentration in mg/m^3^. | | | | | |
| --- | --- | --- | --- | --- | --- |
| **Model** | **Assumptions** | **Default** | **Trial A** | **Trial B** | **Trial D** |
| TRA- Default only | Defaults only: VP > 10 Pa | 93750 |  |  |  |
| TRA Adjusted | Trial Days: adjusted to weight fraction of VOC, grams used, chamber volume |  | 2032 | 1049 | 1483 |
| EGRET – Default only | Defaults only: VP>10 Pa | 1149 |  |  |  |
| EGRET – Trial Days | Trial Day: adjusted to weight fraction of VOC, grams used, chamber volume |  | 1354 | 699 | 988 |
| ConsExpo- Instantaneous Mode (screening level) | Adjusted MW, VP, use amount, weight fraction, ACH, room volume to match experimental conditions | NA | 161 | 109 | 205 |
| ConsExpo- Constant Rate Mode | As above | NA | 148 | 98 | 177 |
| ConsExpo- Evaporation Mode- Langmuir | As above + Log Kow | NA | 137 | 93 | 160 |
| ConsExpo – Evaporation Mode – Thibedeaux | As above | NA | 55 | 46 | 66 |
| E-FAST Defaults | Defaults: MW, VP, weight fraction of VOC adjusted; others at defaults | 8.3 |  |  |  |
| E-FAST Trial Days | Adjusted MW, VP, weight fraction of VOC, mass used, chamber volume, house volume set 1 m3 above chamber volume, ACH, outside of house other than 2 hour period to span use | NA | 3.9 | 2.6 | 4.9 |
| ART 1.5 Activity only estimate | Adjusted temp, VP, weight fraction, chamber volume, ACH, time spent painting  Full shift (8-hr) estimate where non-exposure period = 0 | NA | 16  (2.3-100) | 16  (2.3-100) | 26  (3.9-170) |
| IH MOD 2.0  Well Mixed Room Model with Constant Emission Rate | Adjusted Contaminant mass emission rate (based on weight fraction, use amount, time spent painting), chamber volume and ACH to match experimental conditions | NA | 118 | 80.6 | 217 |

*TRA*

The European Centre for Ecotoxicology and Toxicology of Chemicals Targeted Risk Assessment (ECETOC TRA) tool is a lowest tier screening level model. Predictions are presented based completely on model defaults (no refinement for specific use conditions) as well as modified to match the day of use conditions as closely as possible [VOC weight fraction, mass of paint used and room volume matched to trial conditions]. Note, this model assumes a low air exchange rate (0.6 ACH) which cannot be adjusted. For this model, MW is not required and a VP resolution of > 10 Pa is sufficient, so the averaging of properties for headspace constituents does not impact the predictions. The exposure scenario used in this analysis was PC9a: Coatings, paints, thinners, removers, Product Subcategory waterborn latex wall paint.

*EGRET*

The European Solvent Industry Group’s Generic Exposure Scenario Risk and Exposure Tool (EGRET) is based upon the TRA but with some refinements. Predictions are presented based completely on model defaults as well as to match the conditions during use [VOC weight fraction, mass of paint used and room volume matched to trial conditions]. This model also assumes a low air exchange rate (0.6 ACH) which cannot be adjusted. For the trial days, using the EGRET equations with the air exchange rate adjusted to the experimental conditions results in the same exposure estimates as ConsExpo in instantaneous mode; this is as expected since the EGRET and TRA assumption is that 100% of the weight fraction being modeled is released instantaneously. Similar to the TRA, the averaging of properties for headspace constituents does not impact the predictions. The exposure scenario used in this analysis was the same as for the ECETOC TRA: PC9a: Coatings, paints, thinners, removers, Product Subcategory waterborne latex wall paint.

*ConsExpo*

This consumer exposure model is from the Institute of the Environment for the Netherlands (RIVM). This model can be run in multiple tiers. The instantaneous mode is the lowest tier and assumes that 100% of the weight fraction is instantaneously released to the air, similar to the approach taken in the TRA and EGRET tools. The next step up is constant mode, which assumes that the VOC weight fraction evaporates at a constant rate over the course of the event time. The highest tier, offering the largest refinement of this model, is based upon evaporation of VOC content over time. Two evaporation algorithms are available for use- Langmuir or Thibodeaux. The scenario used in this analysis was Painting Products – Brush and Roller Paints – Waterborne Wall Paint. The model was run in all modes- instantaneous, constant, evaporation with Langmuir and evaporation with Thibodeaux. In all modes molecular weight, vapor pressure, use amount, weight fraction, air changes per hour and room volume were set to match experimental conditions. For evaporation, an average log Kow was also used.

*E-FAST*

The paint module of USEPA’s Exposure and Fate Assessment Screening Tool is based upon the Wall Paint Exposure Model (WPEM) which has been developed with and evaluated against wall painting data. The E-FAST tool sets the scenario in a multi-zone home. Defaults have the user in the room of use to start with, but then with a default pattern of movement between rooms and also in or out of the house. The scenario used in this analysis was latex paint. The model was run first with minimal adjustments: MW, VP, weight fraction of VOC as per the experimental conditions. Additional adjustments were then made to best mimic the experimental chamber conditions: the room of use was set to the volume of the chamber (24 m3), and the entire house volume was set to 25 m^3^ as it had to be greater than the room of use volume. The person was placed in the room of use for 1 or 2 hours, the smallest hourly increment that would cover the event exposure duration, and outside of the house for the remaining time. Event time in the model was set equal to painting duration in the chamber (event time). Years of use were set equal to lifetime with frequency once per day. Note, E-FAST returns either a Lifetime Average Daily Concentration in mg/m^3^ or potential peak concentration in mg/m^3^. The peak concentrations are included in the table above. An attempt was made to back calculate event exposures bases upon the LADC, which provided similar estimates to the peak exposures (Trial A 3.9 mg/m^3^, Trial B 2.3 mg/m^3^, Trial D: 4.09 mg/m^3^) but had the additional uncertainty of event durations not directly matching time in the room. The unmanipulated peak concentrations were therefore used.

*Well-Mixed Box Model (using IH MOD 2.0)*

The Well-Mixed Box Model (also referred to as the well mixed room or one box model) is a simple model that has been used in occupational hygiene to estimate potential exposures. This model assumes that the contaminant is instantaneously mixed, meaning nearly equal concentrations throughout the room. For this study, the simplest version of this model, the well mixed room with constant emission rate was used (available in IHMOD 2.0, a mathematical modeling Excel spreadsheet used for estimating occupational exposures). Inputs that were modified to match the experimental conditions for this model include the generation rate, ventilation rate, concentration in incoming air, initial concentration in the room, room volume, and duration of exposure. The generation rate (i.e., the contaminant mass emission rate) for each trial was assumed to be constant and calculated based on weight fraction, use amount, and the time spent painting (assumed that all of the VOCs in the paint that was applied to the drywall panels were emitted). Other assumptions for the model included: perfectly mixed room, contaminant concentration at time zero = 0, contaminant concentration in supply air = 0, loss mechanism value = 0%.

*ART (Advanced Reach Tool) 1.5*

The Advanced Reach Tool (ART) is a higher tier tool for inhalation exposures that follows a Bayesian approach, making use of mechanistically modelled estimates of exposure for a range of substance classifications, information on exposure variability from meta-analyses in the literature, and any available exposure measurements (built in library or user measurements). A first estimate of the geometric mean exposure level of a scenario is produced by a mechanistic model. Information from the literature provides the initial estimates of exposure variability between-companies, between-workers, within-workers, and between-company variability. ART calculates an overall distribution for full-shift exposures; however ART can be used for short-term estimates. It is noted that the model is optimized for an 8-hour exposure period. The majority of model inputs were set to match the experimental conditions. The product type and activity selected for the scenarios modeled using ART was “Liquids” (product type), “Spreading of liquid product” subactivity. Temperature, VP, weight fraction, room size (selected 30m3 from the options), and the ACH (selected 10 from listed) were set to match experimental conditions. In addition, the following inputs were selected in the configuration of the activity: primary emission source located in the breathing zone of the worker, spreading of liquid products at 1-3m2/hour, no localized controls, process fully enclosed, and no secondary emission source.

References:

ART 1.5: Advanced Reach Tool. <https://www.advancedreachtool.com/>

ConsExpo: National Institute for Public Health and the Environment, Ministry of Health, Welfare and Sport, The Netherlands (RIVM). <https://www.rivm.nl/en/consexpo>

ECETOC TRA: European Centre for Ecotoxicology and Toxicology of Chemicals Targeted Risk Assessment Tool. <http://www.ecetoc.org/tools/targeted-risk-assessment-tra/>

E-FAST: United States Environmental Protection Agency Exposure and Fate Assessment Screening Tool. <https://www.epa.gov/tsca-screening-tools/e-fast-exposure-and-fate-assessment-screening-tool-version-2014>

EGRET: European Solvent Industry Group Generic Exposure Scenario Risk and Exposure Tool. <https://www.esig.org/reach-ges/consumers/>

WMB: Well-Mixed Box Model with Constant Emission Rate using IHMOD 2.0. American Industrial Hygiene Association Exposure Assessment Strategies Committee. <https://www.aiha.org/get-involved/VolunteerGroups/Pages/Exposure-Assessment-Strategies-Committee.aspx>

**E-FAST, CONSEXPO, ART 1.5 Model Reports (inputs and outputs)**

E-FAST (CEM) Trial A

| CEM Inhalation Exposure Estimates | | | | |
| --- | --- | --- | --- | --- |
| ID Number: tmpcas | | | | |
| Scenario: Latex Paint | | Population: Adult | | |
| Exposure Duration (years) chronic: 78 | | Exposure Duration (days) acute: 1 | | |
|  | |  | | |
|  | |  | | |
|  | Exposure Units | Result | AT (days) |  |
|  | Chronic Cancer |  |  |  |
|  | LADD_pot_ (mg/kg-day) | 3.93e‑02 | 2.85e+04 |  |
|  | LADC_pot_ (mg/m^3^) | 1.77e‑01 | 2.85e+04 |  |
|  | Acute |  |  |  |
|  | ADR_pot_ (mg/kg-day) | 3.94e‑02 | 1 |  |
|  | Cp_pot_ (mg/m^3^) | 3.86e+00 | NA |  |
|  | | | | |
| LADD - Lifetime Average Daily Dose (mg/kg-day) | | LADC - Lifetime Average Daily Concentration (mg/m^3^) | | |
| ADR - Acute Dose Rate (mg/kg-day) | | Cp - Peak Concentration (mg/m^3^) | | |
|  |  | | |  |

Note: 75 years = 2.738e+04 days pot - potential dose

Note: The general Agency guidance for assessing short-term, infrequent events (for most chemicals, an exposure of less than 24 hours that occurs no more frequently than monthly) is to treat such events as independent, acute exposures rather than as chronic exposure. (Methods for Exposure-Response Analysis for Acute Inhalation Exposure to Chemicals (External Review Draft). EPA/600/R-98/051. April 1998)

E-FAST (CEM) Trial B

| CEM Inhalation Exposure Estimates | | | | |
| --- | --- | --- | --- | --- |
| ID Number: tmpcas | | | | |
| Scenario: Latex Paint | | Population: Adult | | |
| Exposure Duration (years) chronic: 78 | | Exposure Duration (days) acute: 1 | | |
|  | |  | | |
|  | |  | | |
|  | Exposure Units | Result | AT (days) |  |
|  | Chronic Cancer |  |  |  |
|  | LADD_pot_ (mg/kg-day) | 1.77e‑02 | 2.85e+04 |  |
|  | LADC_pot_ (mg/m^3^) | 7.97e‑02 | 2.85e+04 |  |
|  | Acute |  |  |  |
|  | ADR_pot_ (mg/kg-day) | 1.77e‑02 | 1 |  |
|  | Cp_pot_ (mg/m^3^) | 2.62e+00 | NA |  |
|  | | | | |
| LADD - Lifetime Average Daily Dose (mg/kg-day) | | LADC - Lifetime Average Daily Concentration (mg/m^3^) | | |
| ADR - Acute Dose Rate (mg/kg-day) | | Cp - Peak Concentration (mg/m^3^) | | |
|  |  | | |  |

Note: 75 years = 2.738e+04 days pot - potential dose

Note: The general Agency guidance for assessing short-term, infrequent events (for most chemicals, an exposure of less than 24 hours that occurs no more frequently than monthly) is to treat such events as independent, acute exposures rather than as chronic exposure. (Methods for Exposure-Response Analysis for Acute Inhalation Exposure to Chemicals (External Review Draft). EPA/600/R-98/051. April 1998)

E-FAST (CEM) Trial D

| CEM Inhalation Exposure Estimates | | | | |
| --- | --- | --- | --- | --- |
| ID Number: tmpcas | | | | |
| Scenario: Latex Paint | | Population: Adult | | |
| Exposure Duration (years) chronic: 78 | | Exposure Duration (days) acute: 1 | | |
|  | |  | | |
|  | |  | | |
|  | Exposure Units | Result | AT (days) |  |
|  | Chronic Cancer |  |  |  |
|  | LADD_pot_ (mg/kg-day) | 3.22e‑02 | 2.85e+04 |  |
|  | LADC_pot_ (mg/m^3^) | 1.45e‑01 | 2.85e+04 |  |
|  | Acute |  |  |  |
|  | ADR_pot_ (mg/kg-day) | 3.22e‑02 | 1 |  |
|  | Cp_pot_ (mg/m^3^) | 4.86e+00 | NA |  |
|  | | | | |
| LADD - Lifetime Average Daily Dose (mg/kg-day) | | LADC - Lifetime Average Daily Concentration (mg/m^3^) | | |
| ADR - Acute Dose Rate (mg/kg-day) | | Cp - Peak Concentration (mg/m^3^) | | |
|  |  | | |  |

Note: 75 years = 2.738e+04 days pot - potential dose

Note: The general Agency guidance for assessing short-term, infrequent events (for most chemicals, an exposure of less than 24 hours that occurs no more frequently than monthly) is to treat such events as independent, acute exposures rather than as chronic exposure. (Methods for Exposure-Response Analysis for Acute Inhalation Exposure to Chemicals (External Review Draft). EPA/600/R-98/051. April 1998)

**ConsExpo Input and Output**

| Name | wallpaint |  |  |
| --- | --- | --- | --- |
| CASNumber |  |  |  |
| Molecular weight | 119 | g/mol |  |
| KOW | 2.1 | 10Log |  |
| Product |  |  |  |
| Name | wallpaint1 | |  |
| Weight fraction substance | 0.02 |  |  |
| Population |  |  |  |
| Name |  |  |  |
| Body weight |  |  |  |
| Scenario WPn06constr |  |  |  |
| Frequency | 2 | per year |  |
| Description |  |  |  |
| Inhalation |  |  |  |
| Exposure model | Exposure to vapour - Constant rate | | |
| Exposure duration | 51 | minute |  |
| Product in pure form | No |  |  |
| Molecular weight matrix | 120 | g/mol |  |
| The product is used in dilution | No |  |  |
| Product amount | 1780 | g |  |
| Weight fraction substance | 0.02 |  |  |
| Room volume | 24 | m³ |  |
| Ventilation rate | 8.5 | per hour |  |
| Inhalation rate |  |  |  |
| Emission duration | 51 | minute |  |
| Limit concentration to saturated air concentration | No |  |  |
| Absorption model | n.a. |  |  |
| Dermal |  |  |  |
| Exposure model | Direct contact - Constant rate | | |
| Exposed area |  |  |  |
| Weight fraction substance | 0.02 |  |  |
| Contact rate | 30 | mg/min |  |
| Release duration | 120 | minute |  |
| Absorption model | n.a. |  |  |
| Oral |  |  |  |
| Exposure model | n.a. |  |  |
| Absorption model | n.a. |  |  |
| Results for scenario WPn06constr |  |  |  |
| Inhalation |  |  |  |
| Mean event concentration | 177 | mg/m³ |  |
| Peak concentration (TWA 15 min) | 205 | mg/m³ |  |
| Mean concentration on day of exposure | 6.27 | mg/m³ |  |
| Year average concentration | 0.0343 | mg/m³ |  |
| External event dose |  |  |  |
| External dose on day of exposure |  |  |  |
| Dermal |  |  |  |
| Dermal load |  |  |  |
| External event dose |  |  |  |
| External dose on day of exposure |  |  |  |
| Scenario WPn06evapLrainc |  |  |  |
| Frequency |  |  |  |
| Description |  |  |  |
| Inhalation |  |  |  |
| Exposure model | Exposure to vapour - Evaporation | | |
| Exposure duration | 51 | minute |  |
| Product in pure form | No |  |  |
| Molecular weight matrix | 120 | g/mol |  |
| The product is used in dilution | No |  |  |
| Product amount | 1780 | g |  |
| Weight fraction substance | 0.02 |  |  |
| Room volume | 24 | m³ |  |
| Ventilation rate | 8.5 | per hour |  |
| Inhalation rate |  |  |  |
| Application temperature | 25 | °C |  |
| Vapour pressure | 2.01E+03 | Pa |  |
| Molecular weight | 119 | g/mol |  |
| Mass transfer coefficient | 207000 | m/hr |  |
| Release area mode | Increasing |  |  |
| Release area | 16598 | cm² |  |
| Application duration | 51 | minute |  |
| Absorption model | n.a. |  |  |
| Dermal |  |  |  |
| Exposure model | n.a. |  |  |
| Absorption model | n.a. |  |  |
| Oral |  |  |  |
| Exposure model | n.a. |  |  |
| Absorption model | n.a. |  |  |
| Results for scenario WPn06evapLrainc | |  |  |
| Inhalation |  |  |  |
| Mean event concentration | 160 | mg/m³ |  |
| Peak concentration (TWA 15 min) | 184 | mg/m³ |  |
| Mean concentration on day of exposure | |  |  |
| Year average concentration |  |  |  |
| External event dose |  |  |  |
| External dose on day of exposure |  |  |  |
| Scenario WPn06evapTrainc |  |  |  |
| Frequency |  |  |  |
| Description |  |  |  |
| Inhalation |  |  |  |
| Exposure model | Exposure to vapour - Evaporation | | |
| Exposure duration | 51 | minute |  |
| Product in pure form | No |  |  |
| Molecular weight matrix | 120 | g/mol |  |
| The product is used in dilution | No |  |  |
| Product amount | 1780 | g |  |
| Weight fraction substance | 0.02 |  |  |
| Room volume | 24 | m³ |  |
| Ventilation rate | 8.5 | per hour |  |
| Inhalation rate |  |  |  |
| Application temperature | 25 | °C |  |
| Vapour pressure | 2.01E+03 | Pa |  |
| Molecular weight | 119 | g/mol |  |
| Mass transfer coefficient | 16 | m/hr |  |
| Release area mode | Increasing |  |  |
| Release area | 16598 | cm² |  |
| Application duration | 51 | minute |  |
| Absorption model | n.a. |  |  |
| Dermal |  |  |  |
| Exposure model | n.a. |  |  |
| Absorption model | n.a. |  |  |
| Oral |  |  |  |
| Exposure model | n.a. |  |  |
| Absorption model | n.a. |  |  |
| Results for scenario WPn06evapTrainc | |  |  |
| Inhalation |  |  |  |
| Mean event concentration | 66.1 | mg/m³ |  |
| Peak concentration (TWA 15 min) | 111 | mg/m³ |  |
| Mean concentration on day of exposure | |  |  |
| Year average concentration |  |  |  |
| External event dose |  |  |  |
| External dose on day of exposure |  |  |  |
| Scenario WPn06inst |  |  |  |
| Frequency | 2 | per year |  |
| Description |  |  |  |
| Inhalation |  |  |  |
| Exposure model | Exposure to vapour - Instantaneous release | | |
| Exposure duration | 51 | minute |  |
| Product in pure form | No |  |  |
| Molecular weight matrix | 120 | g/mol |  |
| The product is used in dilution | No |  |  |
| Product amount | 1780 | g |  |
| Weight fraction substance | 0.02 |  |  |
| Room volume | 24 | m³ |  |
| Ventilation rate | 8.5 | per hour |  |
| Inhalation rate |  |  |  |
| Limit concentration to saturated air concentration | No |  |  |
| Absorption model | n.a. |  |  |
| Dermal |  |  |  |
| Exposure model | Direct contact - Constant rate | | |
| Exposed area |  |  |  |
| Weight fraction substance | 0.02 |  |  |
| Contact rate | 30 | mg/min |  |
| Release duration | 120 | minute |  |
| Absorption model | n.a. |  |  |
| Oral |  |  |  |
| Exposure model | n.a. |  |  |
| Absorption model | n.a. |  |  |
| Results for scenario WPn06inst |  |  |  |
| Inhalation |  |  |  |
| Mean event concentration | 205 | mg/m³ |  |
| Peak concentration (TWA 15 min) | 615 | mg/m³ |  |
| Mean concentration on day of exposure | 7.27 | mg/m³ |  |
| Year average concentration | 0.0398 | mg/m³ |  |
| External event dose |  |  |  |
| External dose on day of exposure |  |  |  |
| Dermal |  |  |  |
| Dermal load |  |  |  |
| External event dose |  |  |  |
| External dose on day of exposure |  |  |  |
| Scenario WPo30constantr |  |  |  |
| Frequency | 2 | per year |  |
| Description |  |  |  |
| Inhalation |  |  |  |
| Exposure model | Exposure to vapour - Constant rate | | |
| Exposure duration | 50 | minute |  |
| Product in pure form | No |  |  |
| Molecular weight matrix | 120 | g/mol |  |
| The product is used in dilution | No |  |  |
| Product amount | 1260 | g |  |
| Weight fraction substance | 0.02 |  |  |
| Room volume | 24 | m³ |  |
| Ventilation rate | 11.5 | per hour |  |
| Inhalation rate |  |  |  |
| Emission duration | 50 | minute |  |
| Limit concentration to saturated air concentration | No |  |  |
| Absorption model | n.a. |  |  |
| Dermal |  |  |  |
| Exposure model | Direct contact - Constant rate | | |
| Exposed area |  |  |  |
| Weight fraction substance | 0.02 |  |  |
| Contact rate | 30 | mg/min |  |
| Release duration | 120 | minute |  |
| Absorption model | n.a. |  |  |
| Oral |  |  |  |
| Exposure model | n.a. |  |  |
| Absorption model | n.a. |  |  |
| Results for scenario WPo30constantr | |  |  |
| Inhalation |  |  |  |
| Mean event concentration | 98.1 | mg/m³ |  |
| Peak concentration (TWA 15 min) | 110 | mg/m³ |  |
| Mean concentration on day of exposure | 3.41 | mg/m³ |  |
| Year average concentration | 0.0187 | mg/m³ |  |
| External event dose |  |  |  |
| External dose on day of exposure |  |  |  |
| Dermal |  |  |  |
| Dermal load |  |  |  |
| External event dose |  |  |  |
| External dose on day of exposure |  |  |  |
| Scenario WPo30evapLrainc |  |  |  |
| Frequency |  |  |  |
| Description |  |  |  |
| Inhalation |  |  |  |
| Exposure model | Exposure to vapour - Evaporation | | |
| Exposure duration | 50 | minute |  |
| Product in pure form | No |  |  |
| Molecular weight matrix | 120 | g/mol |  |
| The product is used in dilution | No |  |  |
| Product amount | 1260 | g |  |
| Weight fraction substance | 0.02 |  |  |
| Room volume | 24 | m³ |  |
| Ventilation rate | 11.5 | per hour |  |
| Inhalation rate |  |  |  |
| Application temperature | 25 | °C |  |
| Vapour pressure | 2.01E+03 | Pa |  |
| Molecular weight | 119 | g/mol |  |
| Mass transfer coefficient | 207000 | m/hr |  |
| Release area mode | Increasing |  |  |
| Release area | 16342 | cm² |  |
| Application duration | 50 | minute |  |
| Absorption model | n.a. |  |  |
| Dermal |  |  |  |
| Exposure model | n.a. |  |  |
| Absorption model | n.a. |  |  |
| Oral |  |  |  |
| Exposure model | n.a. |  |  |
| Absorption model | n.a. |  |  |
| Results for scenario WPo30evapLrainc | |  |  |
| Inhalation |  |  |  |
| Mean event concentration | 92.6 | mg/m³ |  |
| Peak concentration (TWA 15 min) | 104 | mg/m³ |  |
| Mean concentration on day of exposure | |  |  |
| Year average concentration |  |  |  |
| External event dose |  |  |  |
| External dose on day of exposure |  |  |  |
| Scenario WPo30evapTrainc |  |  |  |
| Frequency |  |  |  |
| Description |  |  |  |
| Inhalation |  |  |  |
| Exposure model | Exposure to vapour - Evaporation | | |
| Exposure duration | 50 | minute |  |
| Product in pure form | No |  |  |
| Molecular weight matrix | 120 | g/mol |  |
| The product is used in dilution | No |  |  |
| Product amount | 1259 | g |  |
| Weight fraction substance | 0.02 |  |  |
| Room volume | 24 | m³ |  |
| Ventilation rate | 11.5 | per hour |  |
| Inhalation rate |  |  |  |
| Application temperature | 25 | °C |  |
| Vapour pressure | 2.01E+03 | Pa |  |
| Molecular weight | 119 | g/mol |  |
| Mass transfer coefficient | 16 | m/hr |  |
| Release area mode | Increasing |  |  |
| Release area | 16342 | cm² |  |
| Application duration | 50 | minute |  |
| Absorption model | n.a. |  |  |
| Dermal |  |  |  |
| Exposure model | n.a. |  |  |
| Absorption model | n.a. |  |  |
| Oral |  |  |  |
| Exposure model | n.a. |  |  |
| Absorption model | n.a. |  |  |
| Results for scenario WPo30evapTrainc | |  |  |
| Inhalation |  |  |  |
| Mean event concentration | 46.3 | mg/m³ |  |
| Peak concentration (TWA 15 min) | 74.4 | mg/m³ |  |
| Mean concentration on day of exposure | |  |  |
| Year average concentration |  |  |  |
| External event dose |  |  |  |
| External dose on day of exposure |  |  |  |
| Scenario WPo30inst |  |  |  |
| Frequency | 2 | per year |  |
| Description |  |  |  |
| Inhalation |  |  |  |
| Exposure model | Exposure to vapour - Instantaneous release | | |
| Exposure duration | 50 | minute |  |
| Product in pure form | No |  |  |
| Molecular weight matrix | 120 | g/mol |  |
| The product is used in dilution | No |  |  |
| Product amount | 1259 | g |  |
| Weight fraction substance | 0.02 |  |  |
| Room volume | 24 | m³ |  |
| Ventilation rate | 11.5 | per hour |  |
| Inhalation rate |  |  |  |
| Limit concentration to saturated air concentration | No |  |  |
| Absorption model | n.a. |  |  |
| Dermal |  |  |  |
| Exposure model | Direct contact - Constant rate | | |
| Exposed area |  |  |  |
| Weight fraction substance | 0.02 |  |  |
| Contact rate | 30 | mg/min |  |
| Release duration | 120 | minute |  |
| Absorption model | n.a. |  |  |
| Oral |  |  |  |
| Exposure model | n.a. |  |  |
| Absorption model | n.a. |  |  |
| Results for scenario WPo30inst |  |  |  |
| Inhalation |  |  |  |
| Mean event concentration | 109 | mg/m³ |  |
| Peak concentration (TWA 15 min) | 344 | mg/m³ |  |
| Mean concentration on day of exposure | 3.8 | mg/m³ |  |
| Year average concentration | 0.0208 | mg/m³ |  |
| External event dose |  |  |  |
| External dose on day of exposure |  |  |  |
| Dermal |  |  |  |
| Dermal load |  |  |  |
| External event dose |  |  |  |
| External dose on day of exposure |  |  |  |
| Scenario WPT1constantr |  |  |  |
| Frequency | 2 | per year |  |
| Description |  |  |  |
| Inhalation |  |  |  |
| Exposure model | Exposure to vapour - Constant rate | | |
| Exposure duration | 66 | minute |  |
| Product in pure form | No |  |  |
| Molecular weight matrix | 120 | g/mol |  |
| The product is used in dilution | No |  |  |
| Product amount | 2440 | g |  |
| Weight fraction substance | 0.02 |  |  |
| Room volume | 24 | m³ |  |
| Ventilation rate | 11.5 | per hour |  |
| Inhalation rate |  |  |  |
| Emission duration | 66 | minute |  |
| Limit concentration to saturated air concentration | No |  |  |
| Absorption model | n.a. |  |  |
| Dermal |  |  |  |
| Exposure model | Direct contact - Constant rate | | |
| Exposed area |  |  |  |
| Weight fraction substance | 0.02 |  |  |
| Contact rate | 30 | mg/min |  |
| Release duration | 120 | minute |  |
| Absorption model | n.a. |  |  |
| Oral |  |  |  |
| Exposure model | n.a. |  |  |
| Absorption model | n.a. |  |  |
| Results for scenario WPT1constantr | |  |  |
| Inhalation |  |  |  |
| Mean event concentration | 148 | mg/m³ |  |
| Peak concentration (TWA 15 min) | 161 | mg/m³ |  |
| Mean concentration on day of exposure | 6.78 | mg/m³ |  |
| Year average concentration | 0.0372 | mg/m³ |  |
| External event dose |  |  |  |
| External dose on day of exposure |  |  |  |
| Dermal |  |  |  |
| Dermal load |  |  |  |
| External event dose |  |  |  |
| External dose on day of exposure |  |  |  |
| Scenario WPT1evapLrainc |  |  |  |
| Frequency |  |  |  |
| Description |  |  |  |
| Inhalation |  |  |  |
| Exposure model | Exposure to vapour - Evaporation | | |
| Exposure duration | 66 | minute |  |
| Product in pure form | No |  |  |
| Molecular weight matrix | 120 | g/mol |  |
| The product is used in dilution | No |  |  |
| Product amount | 2440 | g |  |
| Weight fraction substance | 0.02 |  |  |
| Room volume | 24 | m³ |  |
| Ventilation rate | 11.5 | per hour |  |
| Inhalation rate |  |  |  |
| Application temperature | 25 | °C |  |
| Vapour pressure | 2.01E+03 | Pa |  |
| Molecular weight | 119 | g/mol |  |
| Mass transfer coefficient | 207000 | m/hr |  |
| Release area mode | Increasing |  |  |
| Release area | 16600 | cm² |  |
| Application duration | 66 | minute |  |
| Absorption model | n.a. |  |  |
| Dermal |  |  |  |
| Exposure model | n.a. |  |  |
| Absorption model | n.a. |  |  |
| Oral |  |  |  |
| Exposure model | n.a. |  |  |
| Absorption model | n.a. |  |  |
| Results for scenario WPT1evapLrainc | |  |  |
| Inhalation |  |  |  |
| Mean event concentration | 136 | mg/m³ |  |
| Peak concentration (TWA 15 min) | 149 | mg/m³ |  |
| Mean concentration on day of exposure | |  |  |
| Year average concentration |  |  |  |
| External event dose |  |  |  |
| External dose on day of exposure |  |  |  |
| Scenario WPT1evapTrainc |  |  |  |
| Frequency |  |  |  |
| Description |  |  |  |
| Inhalation |  |  |  |
| Exposure model | Exposure to vapour - Evaporation | | |
| Exposure duration | 66 | minute |  |
| Product in pure form | No |  |  |
| Molecular weight matrix | 120 | g/mol |  |
| The product is used in dilution | No |  |  |
| Product amount | 2440 | g |  |
| Weight fraction substance | 0.02 |  |  |
| Room volume | 24 | m³ |  |
| Ventilation rate | 11.5 | per hour |  |
| Inhalation rate |  |  |  |
| Application temperature | 25 | °C |  |
| Vapour pressure | 2.01E+03 | Pa |  |
| Molecular weight | 119 | g/mol |  |
| Mass transfer coefficient | 16 | m/hr |  |
| Release area mode | Increasing |  |  |
| Release area | 16600 | cm² |  |
| Application duration | 66 | minute |  |
| Absorption model | n.a. |  |  |
| Dermal |  |  |  |
| Exposure model | n.a. |  |  |
| Absorption model | n.a. |  |  |
| Oral |  |  |  |
| Exposure model | n.a. |  |  |
| Absorption model | n.a. |  |  |
| Results for scenario WPT1evapTrainc | |  |  |
| Inhalation |  |  |  |
| Mean event concentration | 55.4 | mg/m³ |  |
| Peak concentration (TWA 15 min) | 93.3 | mg/m³ |  |
| Mean concentration on day of exposure | |  |  |
| Year average concentration |  |  |  |
| External event dose |  |  |  |
| External dose on day of exposure |  |  |  |
| Scenario WPT1inst |  |  |  |
| Frequency | 2 | per year |  |
| Description |  |  |  |
| Inhalation |  |  |  |
| Exposure model | Exposure to vapour - Instantaneous release | | |
| Exposure duration | 66 | minute |  |
| Product in pure form | No |  |  |
| Molecular weight matrix | 120 | g/mol |  |
| The product is used in dilution | No |  |  |
| Product amount | 2438 | g |  |
| Weight fraction substance | 0.02 |  |  |
| Room volume | 24 | m³ |  |
| Ventilation rate | 11.5 | per hour |  |
| Inhalation rate |  |  |  |
| Limit concentration to saturated air concentration | No |  |  |
| Absorption model | n.a. |  |  |
| Dermal |  |  |  |
| Exposure model | Direct contact - Constant rate | | |
| Exposed area |  |  |  |
| Weight fraction substance | 0.02 |  |  |
| Contact rate | 30 | mg/min |  |
| Release duration | 120 | minute |  |
| Absorption model | n.a. |  |  |
| Oral |  |  |  |
| Exposure model | n.a. |  |  |
| Absorption model | n.a. |  |  |
| Results for scenario WPT1inst |  |  |  |
| Inhalation |  |  |  |
| Mean event concentration | 161 | mg/m³ |  |
| Peak concentration (TWA 15 min) | 667 | mg/m³ |  |
| Mean concentration on day of exposure | 7.36 | mg/m³ |  |
| Year average concentration | 0.0403 | mg/m³ |  |
| External event dose |  |  |  |
| External dose on day of exposure |  |  |  |
| Dermal |  |  |  |
| Dermal load |  |  |  |
| External event dose |  |  |  |
| External dose on day of exposure |  |  |  |

**ART 1.5 Trial A Details**

| **Details for Activity Painting** | | | | | | | | | | | | | | | | | | | |  |  |  |  |  |  |  |  |
| --- | --- | --- | --- | --- | --- | --- | --- | --- | --- | --- | --- | --- | --- | --- | --- | --- | --- | --- | --- | --- | --- | --- | --- | --- | --- | --- | --- |
|  |  |  | |  | | | | | |  | | |  |  |  | | |  |  |  |  |  |  |  |  |  |  |
| Emission sources: | Near field | 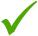   \|  \| \| --- \| \| | |  | | | | | |  | | | Duration (mins): | | | | | 66 |  |  |  |  |  |  |  |  |  |
|  |  |  |  |  | | | | | |  | | |  |  |  | | |  |  |  |  |  |  |  |  |  |  |
|  | Far field |  | |  | | | | | |  | | |  |  |  | | |  |  |  |  |  |  |  |  |  |  |
|  |  |  | |  | | | | | |  | | |  |  |  | | |  |  |  |  |  |  |  |  |  |  |
| **Near-field exposure** | | | | | | | | | | | | | | | | | | | |  |  |  |  |  |  |  |  |
|  |  |  | |  | | | | | |  | | |  |  |  | | |  |  |  |  |  |  |  |  |  |  |
| ***Operational Conditions*** | | | | | | | | | | | | | |  |  | | |  |  |  |  |  |  |  |  |  |  |
|  |  |  | |  | | | | | |  | | |  |  |  | | |  |  |  |  |  |  |  |  |  |  |
| *Substance emission potential* | | |  | | | | | | | | | | | | | | | | | | |  |  |  |  |  |  |
| Substance product type | | | Liquids | | | | | | | | | | | | | | | | | | |  |  |  |  |  |  |
| Process temperature | | | 298 K | | | | | | | | | | | | | | | | | | |  |  |  |  |  |  |
| Vapour pressure | | | 2013.83 Pa | | | | | | | | | | | | | | | | | | |  |  |  |  |  |  |
| Liquid mole fraction | | | 0.02 | | | | | | | | | | | | | | | | | | |  |  |  |  |  |  |
| Activity coefficient | | | 1 | | | | | | | | | | | | | | | | | | |  |  |  |  |  |  |
|  |  |  | |  | |  | |  | | |  | |  | | | |  | | | | | | |  | |  | |
| *Activity emission potential* | | |  | | | | | | | | | | | | | | | | | | |  |  |  |  |  |  |
| Activity class | | | Spreading of liquid products | | | | | | | | | | | | | | | | | | |  |  |  |  |  |  |
| Situation | | | Spreading of liquids at surfaces or work pieces 1.0 - 3.0 m² / hour | | | | | | | | | | | | | | | | | | |  |  |  |  |  |  |
|  |  |  | |  | |  | |  | | |  | |  | | | |  | | | | | | |  | |  | |
| *Surface contamination* | | |  | | | | | | | | | | | | | | | | | | |  |  |  |  |  |  |
| Process fully enclosed? | | | Yes | | | | | | | | | | | | | | | | | | |  |  |  |  |  |  |
|  |  |  | |  | |  | |  | | |  | |  | | | |  | | | | | | |  | |  | |
| *Dispersion* | | |  | | | | | | | | | | | | | | | | | | |  |  |  |  |  |  |
| Work area | | | Indoors | | | | | | | | | | | | | | | | | | |  |  |  |  |  |  |
| Room size | | | 30 m³ | | | | | | | | | | | | | | | | | | |  |  |  |  |  |  |
|  |  |  | |  | |  | |  | | |  | |  | | | |  | | | | | | |  | |  | |
| ***Risk Management Measures*** | | | | |  | |  | |  | | |  | | | |  | | | | | | |  | |  | |  |
|  |  |  | |  | |  | |  | | |  | |  | | | |  | | | | | | |  | |  | |
| *Localised controls* | | |  | | | | | | | | | | | | | | | | | | |  |  |  |  |  |  |
| Primary | | | No localized controls (0.00 % reduction) | | | | | | | | | | | | | | | | | | |  |  |  |  |  |  |
| Secondary | | | No localized controls (0.00 % reduction) | | | | | | | | | | | | | | | | | | |  |  |  |  |  |  |
|  |  |  | |  | |  | |  | | |  | |  | | | |  | | | | | | |  | |  | |
| *Dispersion* | | |  | | | | | | | | | | | | | | | | | | |  |  |  |  |  |  |
| Ventilation rate | | | 10 air changes per hour (ACH) | | | | | | | | | | | | | | | | | | |  |  |  |  |  |  |
|  |  |  | |  | | | | | |  | | |  |  |  | | |  |  |  |  |  |  |  |  |  |  |
| **Predicted exposure levels** | | | | | | | | | | | | | | | | | | | | |  |  |  |  |  |  |  |
| ART predicts air concentrations in a worker's personal breathing zone outside of any Respiratory Protection Equipment (RPE). The use of RPE must be considered separately. | | | | | | | | | | | | | | | | | | | |  |  |  |  |  |  |  |  |
|  |  |  | |  | | | | | |  | | |  |  |  | | |  |  |  |  |  |  |  |  |  |  |
| **Mechanistic model results** | | | | | | | | | | | | | | | | | | | |  |  |  |  |  |  |  |  |
| The predicted 50th percentile full-shift exposure is 16 mg/m³. | | | | | | | | | | | | | | | | | |  |  |  |  |  |  |  |  |  |  |
| The 95% confidence interval is 2.3 mg/m³ to 100 mg/m³. | | | | | | | | | | | | | | | | | |  |  |  |  |  |  |  |  |  |  |

**ART 1.5 Trial B Details**

| **Details for Activity Painting** | | | | | | | | | | |  | |  |  |
| --- | --- | --- | --- | --- | --- | --- | --- | --- | --- | --- | --- | --- | --- | --- |
|  |  |  |  |  |  |  |  | |  | | |  | |  |
| Emission sources: | Near field | 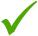   \|  \| \| --- \| \| |  |  | Duration (mins): | | | 50 | |  |  | |  |  |
|  |  |  |  |  |  |  |  | |  | | |  | |  |
|  | Far field |  |  |  |  |  |  | |  | | |  | |  |
|  |  |  |  |  |  |  |  | |  | | |  | |  |
| **Near-field exposure** | | | | | | | | | | |  | |  |  |
| ***Operational Conditions*** | | | | | |  |  | |  | | |  | |  |
|  |  |  |  |  |  |  |  | |  | | |  | |  |
| *Substance emission potential* | | |  | | | | | | | |  |  |  |  |
| Substance product type | | | Liquids | | | | | | | |  |  |  |  |
| Process temperature | | | 298 K | | | | | | | |  |  |  |  |
| Vapour pressure | | | 2013.83 Pa | | | | | | | |  |  |  |  |
| Liquid mole fraction | | | 0.02 | | | | | | | |  |  |  |  |
| Activity coefficient | | | 1 | | | | | | | |  |  |  |  |
|  |  |  |  |  |  |  |  | |  | | |  | |  |
| *Activity emission potential* | | |  | | | | | | | |  |  |  |  |
| Activity class | | | Spreading of liquid products | | | | | | | |  |  |  |  |
| Situation | | | Spreading of liquids at surfaces or work pieces 1.0 - 3.0 m² / hour | | | | | | | |  |  |  |  |
|  |  |  |  |  |  |  |  | |  | | |  | |  |
| *Surface contamination* | | |  | | | | | | | |  |  |  |  |
| Process fully enclosed? | | | Yes | | | | | | | |  |  |  |  |
|  |  |  |  |  |  |  |  | |  | | |  | |  |
| *Dispersion* | | |  | | | | | | | |  |  |  |  |
| Work area | | | Indoors | | | | | | | |  |  |  |  |
| Room size | | | 30 m³ | | | | | | | |  |  |  |  |
| ***Risk Management Measures*** | | | |  |  |  |  | |  | | |  | |  |
|  |  |  |  |  |  |  |  | |  | | |  | |  |
| *Localised controls* | | |  | | | | | | | |  |  |  |  |
| Primary | | | No localized controls (0.00 % reduction) | | | | | | | |  |  |  |  |
| Secondary | | | No localized controls (0.00 % reduction) | | | | | | | |  |  |  |  |
|  |  |  |  |  |  |  |  | |  | | |  | |  |
| *Dispersion* | | |  | | | | | | | |  |  |  |  |
| Ventilation rate | | | 10 air changes per hour (ACH) | | | | | | | |  |  |  |  |
| **Predicted exposure levels** | | | | | | | | | |  |  | |  |  |
|  |  |  |  |  |  |  |  | |  | | |  | |  |
| ART predicts air concentrations in a worker's personal breathing zone outside of any Respiratory Protection Equipment (RPE). The use of RPE must be considered separately. | | | | | | | | | | |  | |  |  |
|  |  |  |  |  |  |  |  | |  | | |  | |  |
| **Mechanistic model results** | | | | | | | | | | |  | |  |  |
| The predicted 50th percentile full-shift exposure is 16 mg/m³. | | | | | | | | | | |  | |  |  |
|  |  |  |  |  |  |  |  | |  | | |  | |  |
| The 95% confidence interval is 2.3 mg/m³ to 100 mg/m³. | | | | | | | | | | |  | |  |  |

**ART 1.5 Trial D Details (ACH=3)***

| **Details for Activity Painting** | | | | | | | | | | | | | | | | | |  |  |  |  |  |  |  |  |  |  |
| --- | --- | --- | --- | --- | --- | --- | --- | --- | --- | --- | --- | --- | --- | --- | --- | --- | --- | --- | --- | --- | --- | --- | --- | --- | --- | --- | --- |
|  |  |  | |  |  | |  |  | |  | | | | |  | | | | | |  |  | |  |  |  |  |
| Emission sources: | Near field | 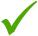   \|  \| \| --- \| \| | |  |  | | Duration (mins): | | | | | 51 | | | |  | |  |  |  |  |  |  |  |  |  |  |
|  |  |  |  |  |  | |  |  | |  | | | | |  | | | | | |  |  | |  |  |  |  |
|  | Far field |  | |  |  | |  |  | |  | | | | |  | | | | | |  |  | |  |  |  |  |
|  |  |  | |  |  | |  |  | |  | | | | |  | | | | | |  |  | |  |  |  |  |
| **Near-field exposure** | | | | | | | | | | | | | | | | | |  |  |  |  |  |  |  |  |  |  |
|  |  |  | |  |  | |  |  | |  | | | | |  | | | | | |  |  | |  |  |  |  |
| ***Operational Conditions*** | | | | | | | |  | |  | | | | |  | | | | | |  |  | |  |  |  |  |
|  |  |  | |  |  | |  |  | |  | | | | |  | | | | | |  |  | |  |  |  |  |
| *Substance emission potential* | | |  | | | | | | | | | | | | | | | |  |  |  |  |  |  |  |  |  |
| Substance product type | | | Liquids | | | | | | | | | | | | | | | |  |  |  |  |  |  |  |  |  |
| Process temperature | | | 298 K | | | | | | | | | | | | | | | |  |  |  |  |  |  |  |  |  |
| Vapour pressure | | | 2013.83 Pa | | | | | | | | | | | | | | | |  |  |  |  |  |  |  |  |  |
| Liquid mole fraction | | | 0.02 | | | | | | | | | | | | | | | |  |  |  |  |  |  |  |  |  |
| Activity coefficient | | | 1 | | | | | | | | | | | | | | | |  |  |  |  |  |  |  |  |  |
|  |  |  | |  |  | |  |  | |  | | | | |  | | | | | |  |  | |  |  |  |  |
| *Activity emission potential* | | |  | | | | | | | | | | | | | | | |  |  |  |  |  |  |  |  |  |
| Activity class | | | Spreading of liquid products | | | | | | | | | | | | | | | |  |  |  |  |  |  |  |  |  |
| Situation | | | Spreading of liquids at surfaces or work pieces 1.0 - 3.0 m² / hour | | | | | | | | | | | | | | | |  |  |  |  |  |  |  |  |  |
|  |  |  | |  |  | |  |  | |  | | | | |  | | | | | |  |  | |  |  |  |  |
| *Surface contamination* | | |  | | | | | | | | | | | | | | | |  |  |  |  |  |  |  |  |  |
| Process fully enclosed? | | | Yes | | | | | | | | | | | | | | | |  |  |  |  |  |  |  |  |  |
|  |  |  | |  |  | |  |  | |  | | | | |  | | | | | |  |  | |  |  |  |  |
| *Dispersion* | | |  | | | | | | | | | | | | | | | |  |  |  |  |  |  |  |  |  |
| Work area | | | Indoors | | | | | | | | | | | | | | | |  |  |  |  |  |  |  |  |  |
| Room size | | | 30 m³ | | | | | | | | | | | | | | | |  |  |  |  |  |  |  |  |  |
|  |  |  | |  |  | |  |  | |  | | | | |  | | | | | |  |  | |  |  |  |  |
| ***Risk Management Measures*** | | | | |  | |  |  | |  | | | | |  | | | | | |  |  | |  |  |  |  |
|  |  |  | |  |  | |  |  | |  | | | | |  | | | | | |  |  | |  |  |  |  |
| *Localised controls* | | |  | | | | | | | | | | | | | | | |  |  |  |  |  |  |  |  |  |
| Primary | | | No localized controls (0.00 % reduction) | | | | | | | | | | | | | | | |  |  |  |  |  |  |  |  |  |
| Secondary | | | No localized controls (0.00 % reduction) | | | | | | | | | | | | | | | |  |  |  |  |  |  |  |  |  |
|  |  |  | |  |  | |  |  | |  | | | | |  | | | | | |  |  | |  |  |  |  |
| *Dispersion* | | |  | | | | | | | | | | | | | | | |  |  |  |  |  |  |  |  |  |
| Ventilation rate | | | 3 air changes per hour (ACH) | | | | | | | | | | | | | | | |  |  |  |  |  |  |  |  |  |
|  |  |  | |  |  | |  |  | |  | | | | |  | | | | | |  |  | |  |  |  |  |
| **Predicted exposure levels** | | | | | |  | | |  | |  | |  |  | | |  | | | | | |  | | |  |  |
|  |  |  | |  |  | |  |  | |  | | | | |  | | | | | |  |  | |  |  |  |  |
| ART predicts air concentrations in a worker's personal breathing zone outside of any Respiratory Protection Equipment (RPE). The use of RPE must be considered separately. | | | | | | | | | | | | | | | | | |  |  |  |  |  |  |  |  |  |  |
|  |  |  | |  |  | |  |  | |  | | | | |  | | | | | |  |  | |  |  |  |  |
| **Mechanistic model results** | | | | | | | | | | | | | | | | | | | |  |  |  |  |  |  |  |  |
| The predicted 50th percentile full-shift exposure is 36 mg/m³. | | | | | | | | | | | | | | | | | |  |  |  |  |  |  |  |  |  |  |
|  |  |  |  |  |  |  |  |  |  |  |  |  |  |  |  |  |  |  |  |  |  |  |  |  |  |  |  |
| The 95% confidence interval is 5.4 mg/m³ to 240 mg/m³. | | | | | | | | | | | | | | | | | |  |  |  |  |  |  |  |  |  |  |

**Note: Trial D ACH=3 and ACH=10 outputs were averaged as the actual chamber ACH=6*

**ART 1.5 Trial 5 Details (ACH=10)***

| **Details for Activity Painting** | | | | | | | | | | | |  |  |  |  |  |  |
| --- | --- | --- | --- | --- | --- | --- | --- | --- | --- | --- | --- | --- | --- | --- | --- | --- | --- |
|  |  |  | |  |  |  |  |  | |  | | | | |  |  |  |
| Emission sources: | Near field | 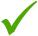   \|  \| \| --- \| \| | |  |  | Duration (mins): | | | 51 | |  |  |  |  |  |  |  |
|  |  |  |  |  |  |  |  |  | |  | | | | |  |  |  |
|  | Far field |  | |  |  |  |  |  | |  | | | | |  |  |  |
|  |  |  | |  |  |  |  |  | |  | | | | |  |  |  |
| **Near-field exposure** | | | | | | | | | | | |  |  |  |  |  |  |
|  |  |  | |  |  |  |  |  | |  | | | | |  |  |  |
| ***Operational Conditions*** | | | | | | |  |  | |  | | | | |  |  |  |
|  |  |  | |  |  |  |  |  | |  | | | | |  |  |  |
| *Substance emission potential* | | |  | | | | | | | | | |  |  |  |  |  |
| Substance product type | | | Liquids | | | | | | | | | |  |  |  |  |  |
| Process temperature | | | 298 K | | | | | | | | | |  |  |  |  |  |
| Vapour pressure | | | 2013.83 Pa | | | | | | | | | |  |  |  |  |  |
| Liquid mole fraction | | | 0.02 | | | | | | | | | |  |  |  |  |  |
| Activity coefficient | | | 1 | | | | | | | | | |  |  |  |  |  |
|  |  |  | |  |  |  |  |  | |  | | | | |  |  |  |
| *Activity emission potential* | | |  | | | | | | | | | |  |  |  |  |  |
| Activity class | | | Spreading of liquid products | | | | | | | | | |  |  |  |  |  |
| Situation | | | Spreading of liquids at surfaces or work pieces 1.0 - 3.0 m² / hour | | | | | | | | | |  |  |  |  |  |
|  |  |  | |  |  |  |  |  | |  | | | | |  |  |  |
| *Surface contamination* | | |  | | | | | | | | | |  |  |  |  |  |
| Process fully enclosed? | | | Yes | | | | | | | | | |  |  |  |  |  |
|  |  |  | |  |  |  |  |  | |  | | | | |  |  |  |
| *Dispersion* | | |  | | | | | | | | | |  |  |  |  |  |
| Work area | | | Indoors | | | | | | | | | |  |  |  |  |  |
| Room size | | | 30 m³ | | | | | | | | | |  |  |  |  |  |
|  |  |  | |  |  |  |  |  | |  | | | | |  |  |  |
| ***Risk Management Measures*** | | | | |  |  |  |  | |  | | | | |  |  |  |
|  |  |  | |  |  |  |  |  | |  | | | | |  |  |  |
| *Localised controls* | | |  | | | | | | | | | |  |  |  |  |  |
| Primary | | | No localized controls (0.00 % reduction) | | | | | | | | | |  |  |  |  |  |
| Secondary | | | No localized controls (0.00 % reduction) | | | | | | | | | |  |  |  |  |  |
|  |  |  | |  |  |  |  |  | |  | | | | |  |  |  |
| *Dispersion* | | |  | | | | | | | | | |  |  |  |  |  |
| Ventilation rate | | | 10 air changes per hour (ACH) | | | | | | | | | |  |  |  |  |  |
|  |  |  | |  |  |  |  |  | |  | | | | |  |  |  |
| **Predicted exposure levels** | | | |  |  |  |  |  | |  | | | | |  |  |  |
|  |  |  | |  |  |  |  |  | |  | | | | |  |  |  |
| ART predicts air concentrations in a worker's personal breathing zone outside of any Respiratory Protection Equipment (RPE). The use of RPE must be considered separately. | | | | | | | | | | | |  |  |  |  |  |  |
|  |  |  | |  |  |  |  |  | |  | | | | |  |  |  |
| **Mechanistic model results** | | | | | | | | | | | |  |  |  |  |  |  |
|  |  |  | |  |  |  |  |  | |  | | | | |  |  |  |
| The predicted 50th percentile full-shift exposure is 16 mg/m³. | | | | | | | | | | | |  |  |  |  |  |  |
| The 95% confidence interval is 2.3 mg/m³ to 100 mg/m³. | | | | | | | | | | | | | |  |  |  |  |

**Note: Trial D ACH=3 and ACH=10 outputs were averaged as the actual chamber ACH=6*
