# Supplementary material for: Exploring the utility of robots in exposure studies
Source: J Expo Sci Environ Epidemiol. 2019 Nov 19;31(4):784–94. doi: 10.1038/s41370-019-0190-x (PMC7234925; doi:10.1038/s41370-019-0190-x)
Supplement: Supplementary file 2 — Supplementary Material 2 [file 41370_2019_190_MOESM2_ESM.docx]

**Exploring the Utility of Robots in Exposure Studies**

Elisabeth Feld-Cook^1^, Rahul Shome^2^, Rosemary Zaleski^3^, Krishnan Mohan^1^, Hristiyan Kourtev^2^, Kostas Bekris^2^, Clifford Weisel^1^, Jennifer Shin*^4^

1. Rutgers University, Environmental and Occupational Health Sciences Institute, Piscataway, NJ 08854

2. PRACSYS Lab, Department of Computer Science, School of Arts and Sciences at Rutgers University, Piscataway, NJ 08854

3. ExxonMobil Biomedical Sciences Inc., Annandale, NJ 08801

4. ExxonMobil Biomedical Sciences Inc., Spring, TX 77389

**Supplemental Material 2**

GC/MS Headspace Analysis of WBP

One gram of paint was pipetted into a 40 mL vial, capped with a PTFE septum, and was kept on the benchtop to equilibrate at room temperature for one hour. Using an air tight syringe, a 250 µl aliquot of the headspace was taken and injected into an HP 6890/5973 GC/MSD running for 50 minutes with a temperature gradient program starting at 35 °C and ending at a final temperature of 300 °C. The GC/MSD injection port was kept at 250 °C, column dimensions were 30 m x 0.25 mm x 0.1 µm, and the MSD was operating under EI scan mode. Compounds were identified by peak analysis of the resulting chromatogram using the Wiley275 library with a 90 % probability criterion in MSD Chemstation software. No individual compound identifications were verified with corresponding standards at this time.

Table S1. Full set of painted area measurements for all drywall used for all trials.

|  | Drywall Location | Height (cm) | Width (cm) | Area (cm^2^) |
| --- | --- | --- | --- | --- |
| Trial A | Right | 60 | 55 | 3300 |
|  | Front Right | 63 | 78 | 4914 |
|  | Front Left | 59 | 78 | 4602 |
|  | Left | 59 | 64 | 3776 |
| Trial B | Right | 60 | 58 | 3480 |
|  | Front Right | 60 | 78 | 4680 |
|  | Front Left | 58 | 78 | 4524 |
|  | Left | 59 | 62 | 3658 |
| Trial C | Right | 60 | 59 | 3540 |
|  | Front Right | 60 | 77 | 4620 |
|  | Front Left | 58 | 77 | 4466 |
|  | Left | 63 | 59 | 3717 |
| Trial D | Right | 59 | 60 | 3540 |
|  | Front Right | 61 | 78 | 4758 |
|  | Front Left | 58 | 78 | 4524 |
|  | Left | 59 | 64 | 3776 |
| Trial E | Right | 59 | 56 | 3304 |
|  | Front Right | 58 | 78 | 4524 |
|  | Front Left | 59 | 78 | 4602 |
|  | Left | 60 | 64 | 3840 |
| Trial F | Right | 59 | 59 | 3481 |
|  | Front Right | 59 | 75 | 4425 |
|  | Front Left | 58 | 77 | 4466 |
|  | Left | 59 | 63 | 3717 |

Figure S1. Background THC analyzer and VOC monitor collection (top) and background temperature (°C) and relative humidity (%) (bottom).

Figure S2. THC analyzer collection for 24 hours starting at the time of painting for trials performed at a higher air exchange rate: A, B, and C.

Figure S3. VOC monitor collection for 8 hours starting at the time of painting for trials performed at a higher air exchange rate: A, B, and C. The top plot is VOC Monitor Right, the middle plot is VOC Monitor Middle, and the bottom plot is VOC monitor Left.

Figure S4. Trial A temperature and humidity during and after the painting session.

Figure S5. Trial B temperature and humidity during and after the painting session.

Figure S6. Trial C temperature and humidity during and after the painting session.

Figure S7. THC analyzer collection for 24 hours starting at the time of painting for trials performed at a lower air exchange rate: D, E, and F.

Figure S8. VOC monitor collection for 8 hours starting at the time of painting for trials performed at a lower air exchange rate: D, E, and F. The top plot is VOC Monitor Right, the middle plot is VOC Monitor Middle, and the bottom plot is VOC monitor Left.

Figure S9. Trial D temperature and humidity during and after the painting session.

Figure S10. Trial E temperature and humidity during and after the painting session.

Figure S11. Trial F temperature and humidity during and after the painting session.
